# SOCIAL WORKERS AS INFORMAL LEGAL INTERMEDIARIES: RESOLVING CONFLICTING RIGHTS IN NURSING HOMES

**DOI:** 10.1093/geroni/igad104.0108

**Published:** 2023-12-21

**Authors:** Angela Perone

**Affiliations:** University of California, Berkeley, Berkeley, California, United States

## Abstract

Conflicting rights arise often in nursing homes among residents, staff, and family. Nursing home social workers sit at a unique nexus of these rights, given their macro, mezzo, and micro-level training. This study employs a multi-method qualitative design with semi-structured staff interviews (n=90) (direct care, mid-level professional, top management), content analysis of long-term care facility policies (n=376), and ethnographic observation of two facilities (n=8 months) for a multi-layered cross-comparative in-depth case study. While social workers represented only a very small number of the overall nursing home workforce, data revealed the overwhelming reliance on social workers to resolve conflicting rights that arose among residents, staff, and family. Certified nursing assistants, nurses, directors, and administrators regularly deferred to social workers via written policies and unwritten practices to resolve a variety of issues, including discrimination concerns by staff, residents, or family, concerns about quality of care and workforce shortage, and concerns about conflicting rights to resident autonomy, dignity, medical decision-making, and safety (e.g., bed rails). Staff at all levels and professions described the emotional labor and unique professional experience that these conflicts required and felt ill-equipped to resolve these issues. While social workers resolved most of these conflicts, they, too, reported feeling ill-prepared for this role and worried about out-of-scope practice. Ultimately, they relied on their social work training in systems-level change, case management, and interpersonal communication to resolve conflicting rights. This presentation will discuss social worker challenges as informal legal intermediaries and opportunities for better support and training.

